# Comprehensive Analysis of the Transcriptome-Wide m6A Methylation in Mouse Pachytene Spermatocytes and Round Spermatids

**DOI:** 10.3389/fgene.2022.832677

**Published:** 2022-03-17

**Authors:** Shihao Hong, Xiaozhong Shen, Jinmei Cheng, Hanyu Tang, Fei Sun

**Affiliations:** Institute of Reproductive Medicine, Medical School of Nantong University, Nantong, China

**Keywords:** m6A methylation, merip sequencing, spermatogenesis, pachytene spermatocytes, round spermatids

## Abstract

Spermatogenesis, an efficient and complex system in male germline development, requires a series of elaborately regulated genetic events in which diploid spermatogonia differentiate into haploid spermatozoa. N6-methyladenosine (m6A) is an important epigenetic RNA modification that occurs during spermatogenesis. ALKBH5 is an m6A eraser and knocking out *Alkbh5* increases the level of total m6A methylation and causes male infertility. In this study, comprehensive analyses of MeRIP-seq and RNA-seq data revealed differences between wild-type (WT) and *Alkbh5* knockout (KO) mice. In pachytene spermatocytes (PA), 8,151 m6A peaks associated with 9,959 genes were tested from WT and 10,856 m6A peaks associated with 10,016 genes were tested from KO mice. In the round spermatids (RO), 10,271 m6A peaks associated with 10,109 genes were tested from WT mice and 9,559 m6A peaks associated with 10,138 genes were tested from KO mice. The peaks were mainly concentrated in the coding region and the stop codon of the GGAC motif. In addition, enrichment analysis showed significant m6A methylation genes in related pathways in spermatogenesis. Furthermore, we conducted joint analyses of the m6A methylome and RNA transcription, suggesting an m6A regulatory mechanism of gene expression. Finally, seven differentially expressed mRNAs from RNA-seq data in both PA and RO were verified using qPCR. Overall, our study provides new information on m6A modification changes between WT and KO in PA and RO, and may provide new insights into the molecular mechanisms of m6A modification in germ cell development and spermatogenesis.

## Introduction

In mammals, spermatogenesis consists of a series of tightly controlled processes, including mitosis, meiosis, and spermiogenesis (Li et al., 2014; Griswold, 2016). The appropriate function of spermatogonial stem cells (SSCs) determines the sustained production of sperm (Oatley and Brinster, 2012). SSCs proliferate through mitosis, and the resulting daughter cells are divided into two types: those that maintain their characteristics for mitosis, and those that divide to form A paired (Apr) and A aligned (Aal) spermatogonial cells (Oatley and Brinster, 2008). The Aal spermatogonia undergo a series of mitotic divisions, followed by differentiation into type B spermatogonia. The type B spermatogonia generate primary spermatocytes which enter meiotic division which includes the pachytene spermatocyte (PA) stage, and homologous chromosome recombination which leads to the formation of haploid round spermatids (RO). Finally, RO transform into mature spermatozoa (Ahmed and de Rooij, 2009; Handel and Schimenti, 2010; O'Donnell et al., 2011; Naro et al., 2017).

N6-methyladenosine (m6A) modification is implicated in the regulation of expression at the post-transcriptional level, including pre-mRNA splicing, mRNA nuclear export, stability, decay, and translation (Wei et al., 1975; Dominissini et al., 2012; Meyer et al., 2012; Wang et al., 2014; Alarcon et al., 2015; Wang et al., 2015). In mammalian cells, m6A is a dynamic and reversible modification process involving writers, erasers, and readers (Jia et al., 2013; Fu et al., 2014; Yue et al., 2015; A. Alemu et al., 2016; Zhao et al., 2017). METTL3, an m6A writer, modulates m6A modification that acts primarily as a major catalytic component, plays a functional role in mice spermatogenesis, as METTL3-deficient mice are infertile (Lin et al., 2017). ALKBH5, an m6A eraser, was recognized as the second m6A demethylase (Ensfelder et al., 2018). Upon deletion of *Alkbh5*, the fertility of male mice was impaired due to apoptosis of pachytene and metaphase-stage spermatocytes and aberrant spermiogenesis. Significantly reduced numbers of PA and RO were observed in *Alkbh5* knockout (KO) mice (Zheng et al., 2013). Another study showed that *Alkbh5* deficiency resulted in aberrant splicing and increased levels of m6A modification in spermatocytes and RO, generated transcripts with a longer 3ʹ-untranslated region (3ʹ-UTR), and shorter transcripts, suggesting that transcripts with longer 3ʹ-UTR have much higher levels of m6A than those with shorter 3ʹ-UTR near the stop codon (Tang et al., 2018). Furthermore, the study showed that spermatogenesis in *Alkbh5* knockout (KO) mice stagnated at the round spermatozoa phase, and occasionally, some sperm were found in the caudal epididymis of KO mice. These KO sperm were malformed and displayed various structural abnormalities. However, currently, the function of m6A methylation in PA and RO is unclear.

In the present study, we aimed to investigate the role of m6A modification, to facilitate future functional studies of mammalian m6A, and to explore the pathogenic mechanism of spermatogenic disorder. To do this, we examined the m6A peaks in the RNA of PA and RO purified from *Alkbh5* KO and wild-type (WT) adult mouse testes using the STA-PUT method. RNA-Seq and MeRIP-Seq were used to uncover the impact of the *Alkbh5* gene on transcriptome-wide m6A methylation levels in mouse spermatogenesis and to find out whether the functional role of m6A methylome varies between different spermatogenic stages. We also identified the molecular functions within the cell type-specific differential methylation peaks in spermatogenesis, which provides a useful reference for further study into underlying mechanisms in male germline development.

## Materials and Methods

### Mice

The animal study was reviewed and approved by the Institutional Review Board of Nantong University (Nantong, China). The *Alkbh5* ^+/−^ mice were a gift from Dr. Yamei Niu, Institute of Basic Medical Sciences, Chinese Academy of Medical Science, and were purchased from The Jackson Laboratory (Bar Harbor, ME). Homozygous *Alkbh5* mice were produced by intercrossing of heterozygous Alkbh5 mice. Mice were housed in a temperature-and humidity-controlled cages with 12-h light/dark cycles. All WT and *Alkbh5* KO mice used in this study were C57BL/6J (B6) mice. Male *Alkbh5* KO mice have been previously reported (Zheng et al., 2013).

### Gross Morphology and Sperm Analysis

Testes and whole epididymis were dissected from 12-week-old WT and *Alkbh5* KO mice, and washed and immersed in phosphate buffered saline (PBS). After weighing the testis and recording its morphology, the cauda epididymitis was punctured several times with a needle, and the sperm was released into a PBS solution at 37°C for 30 min. Sperm motility was determined using computer-assisted sperm analysis (CASA) and the measurements were repeated thrice. Sperm concentration was measured in triplicate using a hemacytometer.

### Isolation of Spermatogenic Cells at Various Stages of Development

PA and RO were obtained from five adult WT and ten adult *Alkbh5* KO mice testes using the STA-PUT method as described previously (Song et al., 2009). Cell purity and morphology were examined using a microscope, fractions containing similar cell types were mixed and centrifuged to collect purified PA and RO. The purity of the PA and RO cell fraction was higher than 90% as determined by the cell morphology analysis, as previously described (Bryant et al., 2013). The above experiments were repeated three times.

### Histology

Testes and epididymis from WT and KO mice were fixed in 4% paraformaldehyde solution, dehydrated through gradient mixtures of ethyl alcohol and water, cleared by passing through xylene, embedded in paraffin, and sectioned to 5 μm-thickness. The sections were stained with haematoxylin and eosin (HE) solution. Finally, the micrographs were obtained using a light microscope (Leica, DM 2000, Germany).

### Western Blot Analysis

Total protein lysates were extracted from whole testes of WT and KO mice using RIPA lysis buffer. The Pierce BCA Protein Assay Kit (P0012, Beyotime) was used to determine the protein concentration. Protein lysates (30 μg) were resuspended in sodium dodecyl sulphate (SDS) sample buffer, subjected to electrophoresis on a 10% SDS-polyacrylamide gel, and then transferred onto nitrocellulose membranes. The membranes were blocked with skimmed milk at a 5% concentration and incubated at 20°C for 1 h. Next, the membrane was incubated with anti-Alkbh5 (1:500, HPA007196; Sigma) and anti-β-tubulin (1:3000, ab108342; Abcam) antibodies overnight at 4°C, washed in PBS-T the next day, incubated with goat anti-rabbit HRP (1:5000, ab205718; Abcam) at 20°C for 1 h. Proteins were finally visualized using an Odyssey infrared imaging system (Li-Cor Biosciences).

### Quantitative Real-Time PCR Assay

Total RNA was extracted from the testicular tissues of WT and KO mice using TRIzol reagent (Invitrogen, Carlsbad, CA, USA). A total of 1 μg of total RNA was reverse transcribed using the HiScript Ⅱfirst Strand cDNA Synthesis Kit (Vazyme Biotech Co., Ltd). Real-time PCR was performed using TB Green™ Premix Ex Taq™ II (TakaRa Biotechnology, Kyoto, Japan) according to the manufacturer’s instructions. All primers for genes were synthesized by Genewiz and are shown in Supplementary Table S1. The relative abundance of mRNA was calculated using the 2^−ΔΔCt^ method and normalized to β-actin mRNA levels.

### MeRIP Sequencing and RNA Sequencing

Tissue samples were isolated using grinding beads and a high-speed reciprocating shaking device (Tissuelyser-24) in a low-temperature grinding environment, and total RNA from three sets of PA and RO was isolated using TRIzol™ Reagent (cat. No 15596018; Invitrogen). The isolated RNA was treated with DNase I (M0303S; NEB) to remove genomic DNA contamination. Extracted RNA was precipitated in glycogen (25 μg/ml final) and isopropanol at −30°C for 2 h. The pellet was rinsed twice with 70% ethanol, dried, and dissolved in ultra-pure water, then the RNA was fragmented. Briefly, 2 μl 10× RNA fragmentation buffer (100 mM Tris-HCl, 100 mM ZnCl2 in nuclease-free water) was added, and 2 μl 0.5 M EDTA was added immediately after 6 min incubation at 70°C to terminate the reaction (Zeng et al., 2018). The fragmented RNA was purified and recovered using the Zymo RNA Clean and Concentrator-5 Kit (cat. no R1013; Zymo Research). A portion of the purified fragmented RNA was saved as an input control. The remainder of purified fragmented RNA was incubated with 2 μg anti-m6A antibody (ABE572; Sigma-Aldrich) in an IP reaction system containing Dynabeads Protein A (10002D; Invitrogen) and Dynabeads Protein G (10004D; Invitrogen) overnight at 4°C with gentle shaking. After magnetic separation of the beads, the supernatant was removed and ×5 precipitation buffer and RNA enzyme inhibitor were added. The reaction proceeded at 4°C for 1–3 h, followed by 2–3 washes with a low salt precipitation buffer and 2–3 washes with a high salt buffer solution. Thereafter, Dynabeads were placed in an elution buffer (200 μl) at 50°C for 30 min to separate out the m6A-antibody immunoprecipitated RNA fragments. The mRNA was purified via phenol-chloroform extraction and ethanol precipitation. The products were removed by ribosomal RNA, first-strand cDNA was synthesized according to the SMART protocol, and fragments were enriched through PCR amplification for cDNA library construction. The magnetic bead library fragments were purified by DNA to extract ultramicro-RNA methylated m6A to detect the library. A Bioptic QseP70 Analyzer was used to inspect the library and determine whether the size distribution of the library conforms to the theoretical size. Lastly, the PE150 sequencing mode was performed on the NovaSeq high-throughput sequencing platform.

### Data Analysis

The bioinformatics data analysis were performed by Guangzhou Epibiotek Co., Ltd. Cutadapt (v2.5) was used to trim adapters and filter for sequences. The remaining sequence reads were aligned to the mouse Ensembl genome GRCm38. We used Hisat2 aligner (v2.1.0) under the parameters: “--rna-strandness RF” (Kim et al., 2015). m6A peak calling was performed using the exomePeak R package (v2.13.2) under parameters: “PEAK_CUTOFF_PVALUE = 0.05, PEAK_CUTOFF_FDR = NA, FRAGMENT_LENGTH = 200” (Meng et al., 2014). Differential m6A peaks were identified using the exomePeak R package under the indicated parameters. GO and REACTOME analyses of the differentially expressed genes (DEGs) were performed using the clusterprofile R package (v3.6.0). The resulting data were visualized using the Guitar R package (v1.16.0) under the parameter “-len 6-RNA”. Homer (V4.10.4) was used to select the m6A peak with a *p*-value < 0.05 for *de novo* motif analysis.

## Results

### *Alkbh5* Knockout Mice Display Impaired Spermatogenesis

**FIGURE 1:**
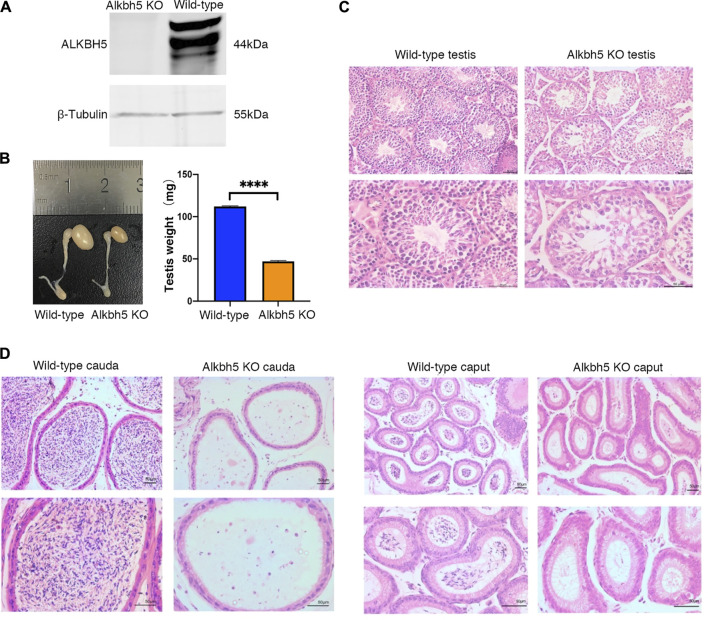
Western blot analysis of the ALKBH5 protein. β-Tubulin was used as the loading control **(A)**. Representative right testes and right epididymis from 12-week-old WT and *Alkbh5* KO mice (left image). Average testis weight from 12-week-old WT and *Alkbh5* KO mice (right image) Bars indicate means ± SEM (n = 10, ****p < 0.0001) **(B)**. Seminiferous tubule architecture of WT and Alkbh5 KO mice were stained with hematoxylin-eosin (H&E) (Scale bars, 50 μm) **(C)**. Cauda and caput epididymis from WT and Alkbh5 KO mice stained with H&E (Scale bars, 50 μm) **(D)**.

Western blot analysis revealed that the ALKBH5 protein was indeed deleted in KO mice (Figure 1A). To investigate the cause of infertility in *Alkbh5* KO mice, we first analysed testicular development from gross morphology and histological perspectives. The shape and mass of the testes in the KO mice were smaller and lower respectively than those in the WT group (Figure 1B). Histological analysis of testes sections revealed that the seminiferous tubules from KO mice contained multiple vacuoles of varying sizes and fewer spermatogenic cells compared to those from WT mice (Figure 1C). Furthermore, caput and cauda epididymis from *Alkbh5*-KO mice showed only a few degenerating immature spermatids or spermatocytes (Figure 1D). To further analyse this, we isolated epididymal spermatozoa from adult WT and KO mice, even though sperm counts in the epididymis of KO mice were low. Using HE staining, we also found that the spermatozoa from KO mice showed various structural abnormalities (Supplementary Figures S1). As expected, KO mice exhibited reduced sperm counts and impaired sperm motility and morphology (Supplementary Figures S2).

### Overview of the m6A Methylation Map in Pachytene Spermatocytes (PA) and Round Spermatids (RO)

**FIGURE 2:**
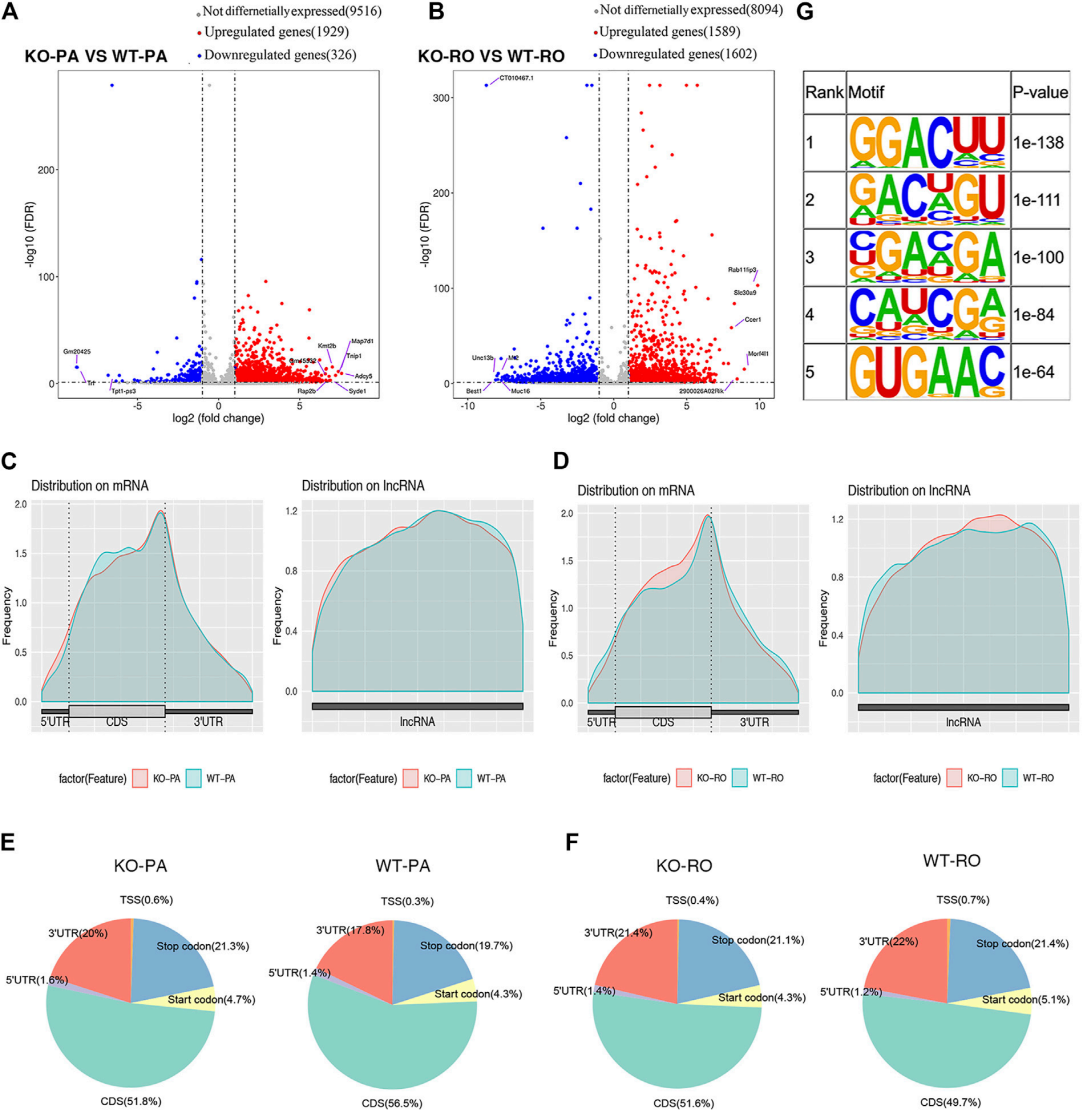
Volcano plot showing differential methylation peaks between WT PA and Alkbh5-KO PA **(A)**. Volcano plot showing differential methylation peaks between WT RO and *Alkbh5*-KO RO **(B)**. m6A peaks were most enriched in the coding sequence near the stop codon on mRNA and the distribution on lncRNA in PA **(C)**. m6A peaks were most enriched in the coding sequence near the stop codon on mRNA and the distribution on lncRNA in RO **(D)**. Distribution of different regions in *Alkbh5*-KO and WT PA **(E)**. Distribution of different regions in *Alkbh5*-KO and WT RO **(F)**. Top five consensus sequence motifs enriched in m6A peaks identified from Alkbh5-KO PA **(G)**.

We performed MeRIP-seq analysis of spermatogenic cells in PA and RO at various stages from *Alkbh5*-KO and WT mice (Meng et al., 2013). *Alkbh5*-KO PA had 1,929 significantly upregulated methylation peaks, and 326 downregulated peaks (|log2FC| > 1 and FDR <0.05). The *Alkbh5*-KO RO had 1,589 significantly upregulated methylation peaks, while 1,602 were downregulated compared with that in the WT RO (Figures 2A,B), suggesting that knocking out *Alkbh5* may have a considerable effect on the upregulation of methylation in PA, while the number of downregulated methylation peaks was much smaller than that of upregulated peaks in PA. However, RO results differ from those of PA where the number of upregulated methylation peaks was found to be similar to that of downregulated methylation peaks. The top 10 altered methylation peaks in PA and RO are presented in Supplementary Table S2. Next, the distribution of methylation peaks in PA from both *Alkbh5*-KO and WT were similar in the 3ʹ-UTR region of mRNA and most parts of the long non-coding (lnc) RNA (Figure 2C). Specifically, methylation peaks in the *Alkbh5*-KO PA indicate a significantly different pattern from peaks in the WT PA, with a relative increase in the number of methylation peaks in the stop codon (21.3 vs. 19.7%), a start codon (4.7% vs. 4.3%), 3ʹ-UTR (20% vs. 17.8%), 5ʹ-UTR (1.6% vs. 1.4%), and TSS (0.6% vs. 0.3%), and a notable decrease in the coding DNA sequences (CDS) (56.5% vs. 51.8%) (Figure 2E). Compared to that in PA, the distribution of different regions in RO differed considerably. The knocking out of *Alkbh5* contributed to a larger change in the distribution of lncRNA in RO than in PA (Figure 2D). The distribution of different regions in RO is conversely different from that in PA. We found that methylation peaks in the *Alkbh5*-KO RO indicate a significantly different pattern from peaks in the WT RO with a relative increase in the number of methylation peaks in the CDS (51.6% vs. 49.7%), 5ʹ-UTR (1.4% vs. 1.2%), and a relative decrease in the 3ʹ-UTR (21.4% vs 22%), TSS (0.4% vs 0.7%), stop codon (21.1% vs 21.4%), and start codon (4.3% vs. 5.1%) (Figure 2F). In addition, the GGAC motif was highly enriched within m6A sites in PA and RO (Figure 2G).

### Differential Methylation Peaks are Enriched in Pathways Related to Spermatogenesis

**FIGURE 3:**
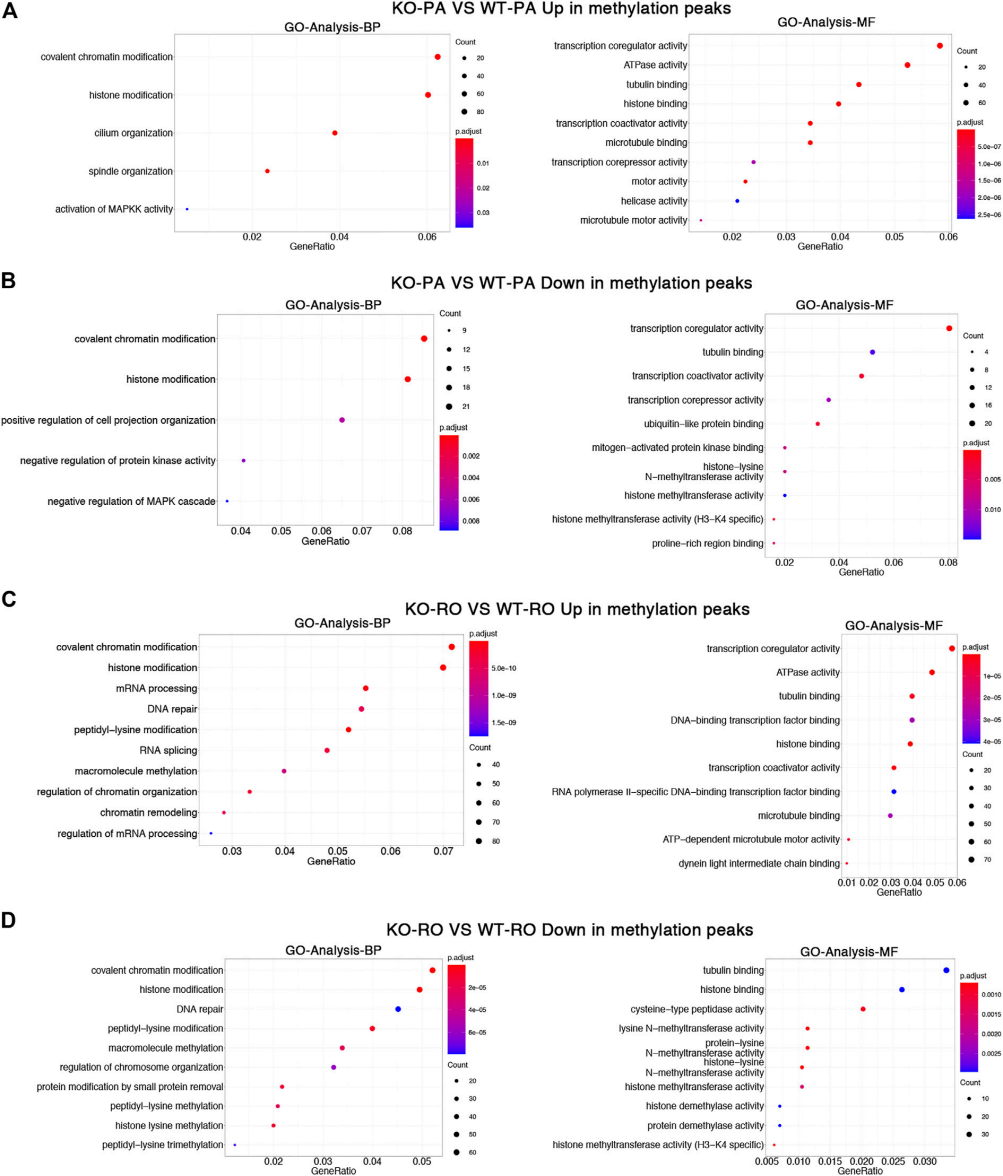
Differentially methylated mRNAs analysis. GO enrichment analysis of upregulated **(A)** and downregulated **(B)** methylated mRNAs between *Alkbh5*-KO PA and WT PA. GO enrichment analysis of upregulated **(C)** and downregulated **(D)** methylated mRNAs between *Alkbh5*-KO RO and WT RO.

To investigate the biological significance of m6A differences between *Alkbh5*-KO and WT spermatogenic cells, GO and REACTOME pathway analyses for differentially methylation peaks were performed (Ashburner et al., 2000; Draghici et al., 2007). GO analysis can be divided into two parts: biological processes (BP) and molecular functions (MF). First, we examined the differences between *Alkbh5*-KO PA and WT PA. We found that both upregulated and downregulated methylation peaks were closely related to covalent chromatin modification and histone modification. Moreover, we found that upregulated methylation peaks are linked with activation of MAPK activity and that downregulated methylation peaks are linked with negative regulation of the MAPK cascade (Figures 3A,B). Next, we analysed the differences between RO from *Alkbh5*-KO and WT. We found that the mechanism of *Alkbh5* in RO may be little different from that in PA, as some upregulated and downregulated methylation peaks are also related to chromatin modification and histone modification (Figures 3C,D). We performed REACTOME-pathway analysis to further study the potential reason for the reproduction dysfunction of *Alkbh5*-KO mice. In the comparison between *Alkbh5*-KO and WT PA, we found that both the upregulated and downregulated methylation peaks were related to chromatin modifying enzymes and chromatin organization. Moreover, we found that the upregulated methylation peaks were related to the M phase. Comparing *Alkbh5*-KO and WT RO, we found that the upregulated methylation peaks are more related to mitotic prometaphase and that the downregulated methylation peaks are more related to RHO GTPase cycle (Supplementary Figures S3).

### Overview of Transcriptome Profiles Revealed Important Mechanisms in a Spermatogenetic Malfunction

**FIGURE 4:**
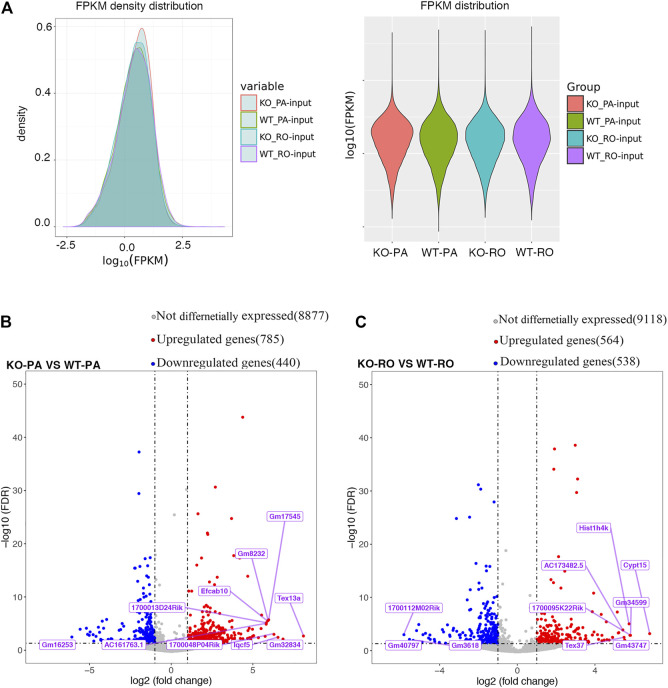
Differential gene analysis FPKM density distribution **(A)**. Volcano plot showing differential genes between *Alkbh5*-KO PA and WT PA **(B)**, *Alkbh5*-KO RO and WT RO **(C)**.

**FIGURE 5:**
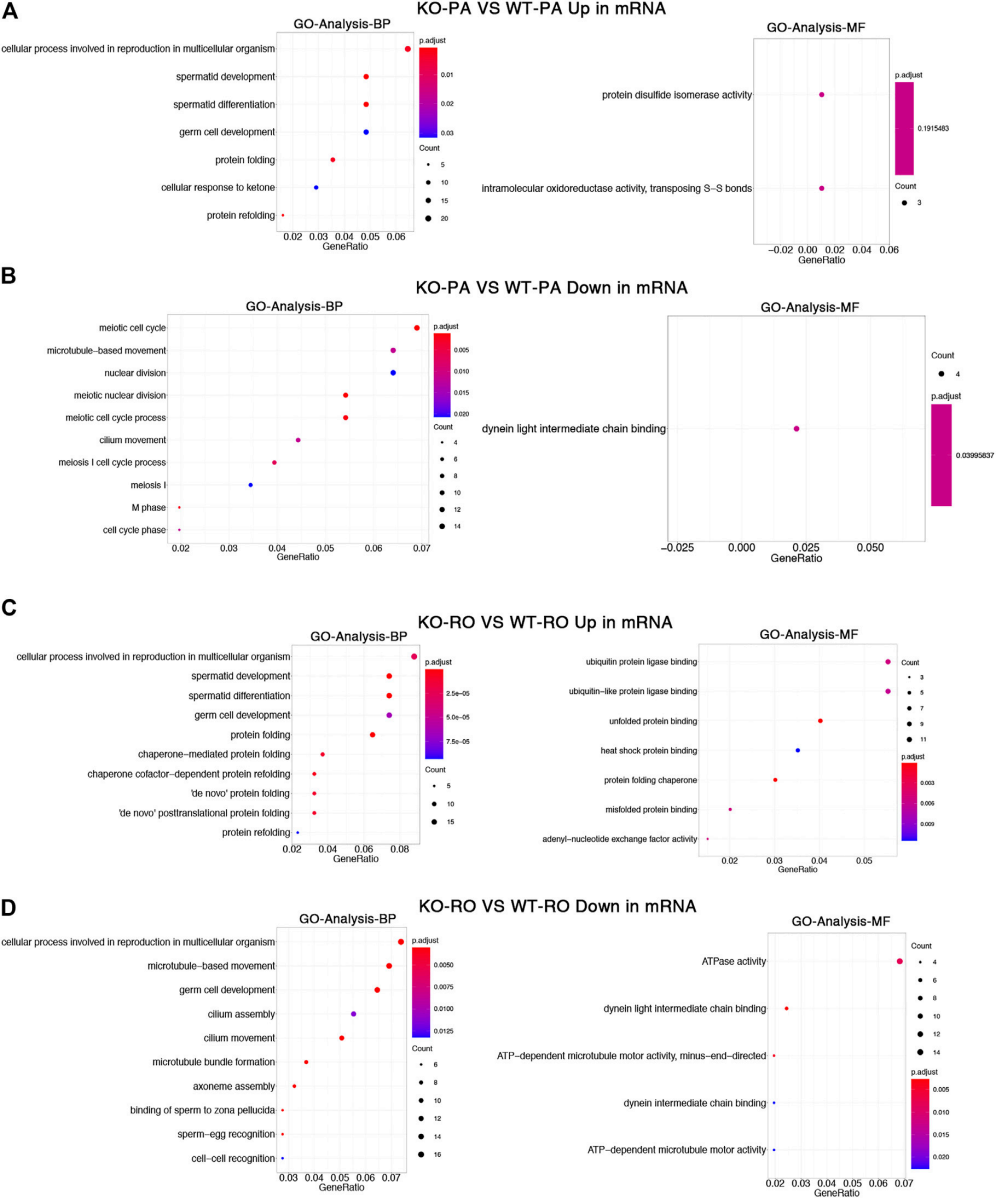
GO enrichment analysis of upregulated **(A)** and downregulated **(B)** genes between *Alkbh5*-KO PA and WT PA. GO enrichment analysis of upregulated **(C)** and downregulated **(D)** genes between *Alkbh5*-KO RO and WT RO.

Using the datasets from RNA-seq results (MeRIP-seq input library), we compared the transcriptome profiles of *Alkbh5*-KO with WT spermatogenic cells. We found no remarkable difference in the density and distribution of expression levels between these samples, which could comprehensively indicate that the sequencing result is reliable (Figure 4A). We found 785 upregulated and 440 downregulated genes in PA (|log2FC| > 1 and FDR <0.05) using the R package DESeq2. We also found 564 upregulated genes and 539 downregulated genes in the RO. The top 10 differentially expressed genes in PA and RO are presented in Supplementary Table S3. In brief, the number of upregulated genes in *Alkbh5*-KO PA was much greater than that in WT PA. However, the number of upregulated genes in *Alkbh5*-KO RO is similar to that in WT RO (Figures 4B,C). Thereafter, we performed GO analysis of the DEGs between the *Alkbh5*-KO and WT spermatogenic cells. GO analysis can be divided into two parts: BP and MF. First, we considered the DEGs between the *Alkbh5*-KO PA and WT PA. From the BP results, we found that these DEGs are closely related to reproduction processes, such as spermatid differentiation, spermatid development and germ cell development. In addition, the upregulated genes are more related to spermatogenesis and the downregulated genes are more related to the meiotic cycle. From this, it may be speculated that knocking out *Alkbh5* may cause damage to the reproductive functions of mice. From the MF results, we found that the upregulated genes were linked with protein disulphide isomerase activity and that the downregulated genes were linked with dynein light intermediate chain binding (Figures 5A,B). Next, we compared the DEGs closely related to reproduction processes between *Alkbh5*-KO RO and WT RO. However, we found that the upregulated genes were more related to protein binding and that the downregulated genes were more related to ATPase activity (Figures 5C,D). Additionally, we screened out DEGs and performed REACTOME enrichment analysis in PA and RO. In the PA and RO, we found that both the downregulated genes were related to fertilization and reproduction. Moreover, we found that the upregulated genes were related to the M phase. Comparing *Alkbh5*-KO and WT RO, we found that the upregulated genes are more related to stress responses (Supplementary Figure S4).

### The Expression of Genes Related to Spermatogenesis is Markedly Altered in *Alkbh5* KO

**FIGURE 6:**
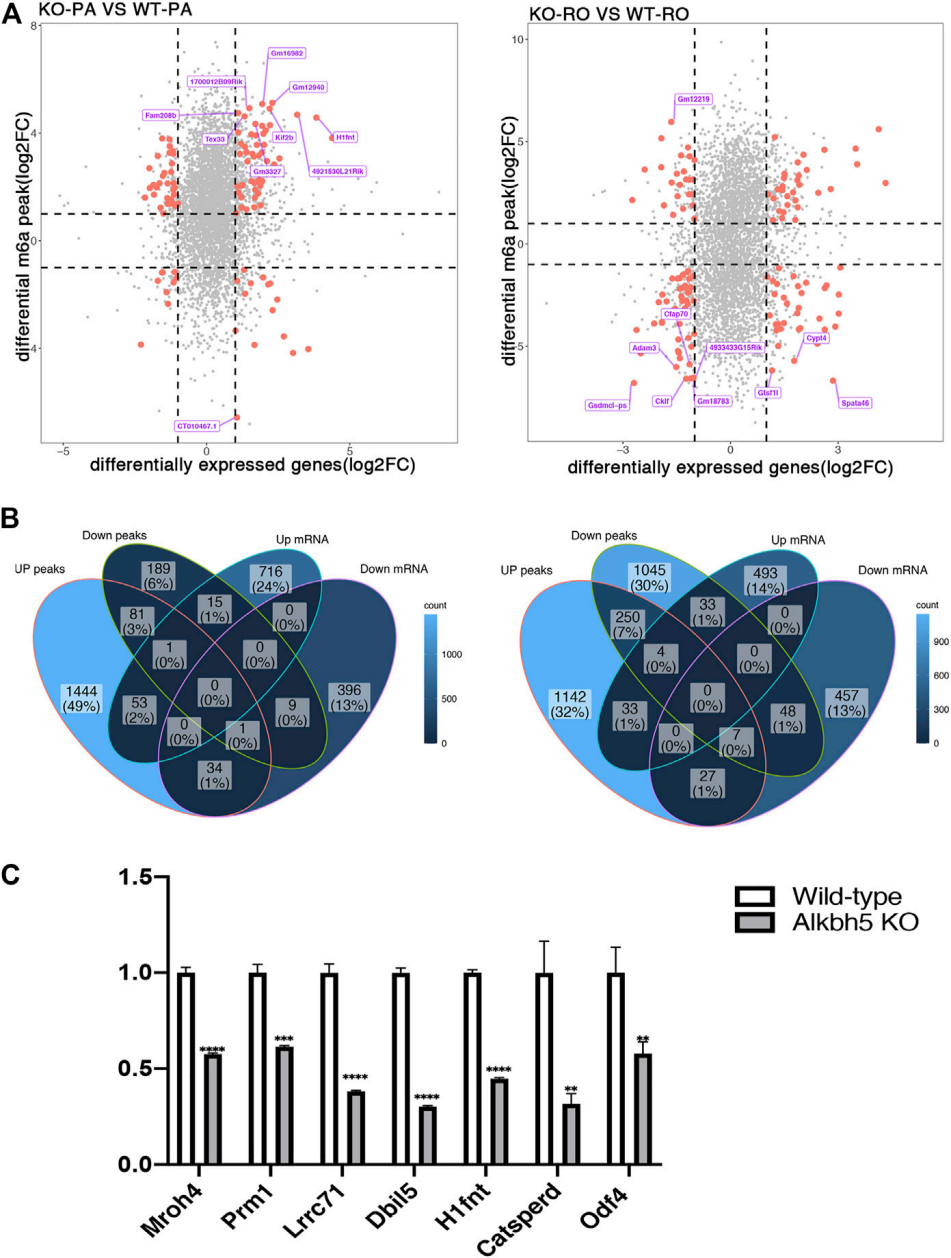
Association analysis of the genes with significant changes in methylation and mRNA expression level in PA and RO **(A)**. Venn diagrams showed the intersection of upregulated and downregulated methylation peaks and mRNAs of PA and RO respectively **(B)**. mRNA expression level of seven genes in *Alkbh5*-KO and WT mice Testicular tissue detected by qPCR (****p < 0.0001, ***p = 0.0001, **p < 0.01.) **(C)**.

To further investigate the influence of *Alkbh5* on spermatogenesis, we performed an association analysis of the genes with significant changes in methylation and mRNA expression levels. The results were divided into four parts: hypermethylated-down, hypermethylated-up, hypomethylated-down, and hypomethylated-up. Figure 6A shows the top 10 differentially methylated genes. To discover the relationship between differential peaks and genes, we counted the number of upregulated and downregulated mRNAs and peaks in KO in PA and RO and calculated their intersection. We found a total of 54 upregulated overlapped genes and 10 downregulated overlapped genes in PA. However, in RO, 37 genes were upregulated, and 55 were downregulated. This revealed the relatively large differences between PA and RO (Figure 6B). Next, we selected the top 20 differential protein-coding genes in *Alkbh5*-KO PA and WT PA (Table 1), as well as in *Alkbh5*-RO and WT RO (Table 2). Thereafter, we intersected the 20 genes in each table. We found that among the top 20 DEGs in each table, there were approximately 11 genes that appeared in both comparisons. We found that these genes were related to the development of spermatogenesis. For this reason, we used qPCR to discover the comprehensive changes in these genes, and whether they exist in the whole testis. We found that *Mroh4, Prm1, Lrrc7, Dbil5, H1fnt, Catsperd*, and *Odf4* were greatly downregulated in the testes in the *Alkbh5* KO (Figure 6C).

**TABLE 1:** The top 20 differential protein-coding genes in *Alkbh5*-KO PA and WT PA.

Gene name	log2FC	Pvalue	FDR
Tnp1	2.28424784	1.24E-160	3.13E-157
mt-Co1	1.26260874	5.59E-124	1.13E-120
Mroh4	−1.9709482	5.52E-41	5.07E-38
Prm1	2.70151334	3.35E-34	2.25E-31
mt-Nd1	0.9318772	9.57E-34	6.04E-31
Lrrc71	−1.9803622	6.09E-33	3.62E-30
BC048507	3.69253326	3.88E-28	1.78E-25
Dbil5	2.21814273	2.06E-25	8.67E-23
H1fnt	3.82950811	5.06E-21	1.70E-18
Ccdc39	−1.2929	1.21E-20	3.70E-18
Fam209	4.18982844	1.60E-20	4.75E-18
Gm5152	1.8510998	1.61E-20	4.66E-18
Prm2	1.56916015	3.43E-19	9.11E-17
Septin12	−1.9199403	6.93E-19	1.71E-16
Gm10800	−2.2059199	1.44E-18	3.38E-16
Catsperd	−2.2408398	1.53E-18	3.52E-16
Ybx2	−1.5076017	5.18E-17	1.03E-14
Tdrd6	−1.3999835	9.60E-17	1.87E-14
Cox8c	2.81876864	9.63E-17	1.84E-14
Odf4	−2.0557199	6.36E-16	1.15E-13

**TABLE 2:** The top 20 differential protein-coding genes in *Alkbh5*-KO RO and WT RO.

Gene name	log2FC	Pvalue	FDR
Tnp1	2.22973416	0	0
Prm1	2.47054444	6.32E-77	9.22E-74
Tnp2	2.98237914	2.35E-42	2.41E-39
Prm2	1.91387341	1.20E-41	1.12E-38
Akap12	1.87493257	9.53E-38	7.50E-35
Lrrc71	−2.0063512	8.92E-35	6.08E-32
Mroh4	−1.8845513	7.02E-34	4.48E-31
Septin12	−2.4637114	1.79E-28	8.30E-26
mt-Cytb	−0.5893197	4.38E-22	1.54E-19
BC048507	2.12079674	6.04E-21	2.06E-18
Mif4gd	−2.1277755	1.19E-19	3.67E-17
Dbil5	1.4354139	3.16E-19	9.23E-17
Leng8	−1.4150108	5.03E-19	1.39E-16
Smcp	1.38820679	8.15E-19	2.19E-16
H1fnt	2.44495495	4.53E-18	1.08E-15
Hspa5	1.73388547	1.93E-16	4.20E-14
Odf1	1.85933744	7.57E-16	1.58E-13
Catsperd	−1.9735095	8.63E-16	1.76E-13
4933409G03	−0.7591224	1.46E-15	2.92E-13
Odf4	−1.8239507	8.72E-15	1.62E-12

## Discussion

Modification of m6A demethylases plays a critical role in mammalian spermatogenesis. ALKBH5 was highly expressed in mouse testis as an m6A eraser, and loss of ALKBH5 significantly increased the level of m6A in testicular cells. *Alkbh5*-KO mice show abnormal spermatogenesis and subfertility (Zheng et al., 2013). An independent study suggested that correct splicing of longer 3ʹ-UTR transcripts may require ALKBH5-mediated m6A demethylation (Tang et al., 2018). However, previous studies on *Alkbh5* only focused on the overall changes in spermatogenesis but not on the specific changes in RNA and m6A methylation changes at each stage of spermatogenesis. To address this question, we performed high-throughput sequencing to reveal the m6A landscape in the spermatogenic stages of PA and RO in *Alkbh5-*KO and WT mice. Using MeRIP-seq, we identified that *Alkbh5*-KO PA had 1,929 noticeably upregulated methylation peaks relative to that in the WT PA, while 326 were downregulated. The *Alkbh5*-KO RO had 1,589 noticeably upregulated methylation peaks relative to that in the WT RO, while 1,602 were downregulated. We then used GO and REACTOME pathway enrichment analyses to explore the potential functions of differentially methylated transcripts. Finally, we found a difference in the genes in methylation peaks and synchronous differential expression genes enriched in the spermatogenesis-related pathway.

In this study, since the analytical results of MeRIP-seq include the results of RNA-seq, our analysis reveals the impact of *Alkbh5* on spermatogenesis from the perspective of differences in methylation and gene expression. We found that the effects of *Alkbh5* on PA and RO differed considerably. First, we found that ALKBH5 is a demethylase has different effects on the methylation of transcripts in PA and RO. In PA, we found that the number of significantly upregulated methylation peaks was far larger than that of significantly downregulated methylation peaks in the *Alkbh5* KO, which is consistent with our previous knowledge about *Alkbh5* that the degree of RNA methylation significantly increases in the testes of *Alkbh5* KOs (Tang et al., 2018). However, in RO, we found that in the *Alkbh5* KO, the number of significantly upregulated methylation peaks was very close to the number of significantly downregulated methylation peaks. We found that the distribution of methylation peaks in PA and RO differed considerably. In particular, in the *Alkbh5* knockout, we found that the proportion of methylation peaks distributed in the CDS region of PA increased significantly, while the proportion of methylation peaks distributed in the CDS region of RO reduced. Moreover, lncRNAs, as epigenetic regulators, play an essential role in spermatogenesis (Wichman et al., 2017), such as the definition of sperm parameters (An et al., 2017) and sperm capacitation (Song et al., 2011). Our observations regarding the distribution of methylation peaks of lncRNA, PA, and RO were also affected by knocking out of *Alkbh5*. The expression of lncRNA was characterized by a sharp increase in the number of expression sites and the overall expression level with the development of spermatogenesis, and reached a peak in the round spermatogenesis stage (Davis et al., 2017).

Here, we used GO enrichment analysis to further explore differentially methylation peaks. We found that after *Alkbh5* knockout, both the upregulated and downregulated methylation peaks in PA and RO were closely related to covalent chromatin modification and histone modification. Histone modification influences chromatin structure and function. Previous studies have indicated various histone modifications are involved in the regulation of spermatogenesis (Okada et al., 2007; Liu et al., 2010; Okada et al., 2010; Iwamori et al., 2011), but the role of covalent chromatin modification in spermatogenesis remains unclear. However, recent studies demonstrated that m6A modification activates gene transcription by altering histone modifications and chromatin structure (Wang et al., 2018). In mouse embryonic stem cells, knocking out METTL3 reduced m6A modification levels and stabilized opening of chromatin that facilitates and sustains gene transcription (Liu et al., 2020). Based on our results, we propose the following conjecture: m6A modification level and related binding proteins on the mRNA of spermatogenic cells at PA and RO can together regulate open state of chromatin and the transcription level of downstream genes, which play an important role in the smooth progress of spermatogenesis, and an abnormal regulation can lead to male infertility. Moreover, genes with upregulated m6A-modified sites in PA were related to activation of MAPK activity and downregulated m6A-modified sites were related to the negative regulation of the MAPK cascade. The MAPK cascade plays important roles in male reproductive processes, such as spermatogenesis, sperm maturation, and the acrosome reaction (Li et al., 2009). TCP1, which is involved in the regulation of the MAPK cascade, is involved in the assembly of actin and tubulin filaments (Dun et al., 2012). Actin filaments exist in mammalian reproductive cells and participate in some of the changes that occur during spermatogenesis, such as sperm maturation and capacitation (Howes et al., 2001). Our results revealed that changes in the m6A modifications of the MAPK signalling pathway may affect sperm maturation and capacitation. Taken together, these factors may contribute to fertility in KO mice.

In the DEG analysis, we detected several the DEGs related to many important biological pathways. REACTOME pathway analysis revealed that transcripts related to fertilization and reproduction genes were downregulated in PA and RO during spermatogenesis, showing that the expression of genes related to spermatogenesis in PA and RO both are affected by the increase in methylation level after knockout of the methylase. This further illustrates that m6A methylation is necessary during spermatogenesis. Using MeRIP-seq results, we analysed the differences from the perspective of RNA. The RNA differential analysis results were similar to the methylation results. We found that the number of upregulated genes in PA was significantly greater than that in the downregulated genes in the *Alkbh5* knockout. However, the number of upregulated and downregulated genes in RO was close. In the enrichment analysis, we found that the differential genes of PA and RO are closely related to spermatogenesis. Therefore, this result reveals that knocking out *Alkbh5* affects gene expression in PA and RO. Further molecular experiments should be performed to verify the differentially methylated genes in mouse PA and RO. Next, we found that the top 20 DEGs in *Alkbh5*-KO PA, WT PA, *Alkbh5*-RO, and WT RO were closely related to spermatogenesis. Moreover, after intersecting these genes, we found that more than half of these genes appeared in both comparisons. In the qPCR experiment, we found that transcripts related to spermatogenesis were significantly downregulated because of *Alkbh5* KO. One of these was, *Prm1*, which is a protamine substitute for histones in the chromatin of sperm during the haploid phase of spermatogenesis, compacts sperm DNA into highly condensed, stable, and inactive complexes (Fagerberg et al., 2014). In addition, we found *Odf4*, which encodes a protein that is localized in the outer dense fibres of the tails of mature sperm. This protein is thought to play an important role in the sperm tail (Nakamura et al., 2002). Furthermore, *H1FNT*, which was also downregulated in *Alkbh5* KO, is a germ cell-specific linker histone variant expressed during spermiogenesis, specifically in round and elongating spermatids (Tanaka et al., 2006). In conclusion, *Alkbh5* greatly contributes to the development of spermatogenesis at both the epigenetic and mRNA levels.

While the causes of approximately 50% of human male infertility are unknown, genetic causes, in particular, often remain undiagnosed (Zhang et al., 2011). Our current mouse study provides basis to a genetic cause for male infertility. *Alkbh5* is also widely expressed in the human testis. In humans, *Alkbh5* expression is higher in PA compared with other stages, implying that it affects semen quality and a perturbation in its function increases the risk of male infertility (Landfors et al., 2016). However, throughout evolution, humans have also developed features in spermatogenesis that are distinguishable from those of mice. It is also unknown whether the m6A methylation level and mRNA expression level in PA and RO will be the same in humans as in mice. This study sheds light on possible targets for future research into developing treatments for certain types of human infertility.

In summary, we analysed the differential m6A methylome in PA and RO in *Alkbh5-*KO mice relative to the corresponding WT mice, and demonstrated a strong association between m6A modification and spermatogenesis. Furthermore, in the *Alkbh5* KO, m6A levels were increased, affecting many spermatogenesis-related genes, very likely leading to spermatogenesis disorders. In addition, the data show that the m6A methylation level and mRNA expression level in mouse PA and RO were significantly different; further research is needed to uncover the underlying mechanisms that affect male fertility.

## Data Availability

The datasets presented in this study can be found in online repositories. The names of the repository/repositories and accession number(s) can be found below: BioProject: PRJNA761579, SRA accession: SRP336482, GEO: GSE186217.
