# Corrigendum: Comprehensive analysis of the transcriptome-wide m6A methylation in mouse pachytene spermatocytes and round spermatids

**DOI:** 10.3389/fgene.2022.969985

**Published:** 2022-08-15

**Authors:** Shihao Hong, Xiaozhong Shen, Jinmei Cheng, Hanyu Tang, Fei Sun

**Affiliations:** Institute of Reproductive Medicine, Medical School of Nantong University, Nantong, China

**Keywords:** m6A methylation, merip sequencing, spermatogenesis, pachytene spermatocytes, round spermatids

In the published article, the source of the mice was omitted in **Materials and Methods**, “*Mice*.” The corrected line appears below.

“The *Alkbh5* ^+/−^ mice were a gift from Dr. Yamei Niu, Institute of Basic Medical Sciences, Chinese Academy of Medical Science, and were purchased from The Jackson Laboratory (Bar Harbor, ME).”

In the published article we neglected to add a line in the **Acknowledgments** statement. The updated **Acknowledgments** statement appears below.

“We thank Dr. Yamei Niu from the Institute of Basic Medical Sciences, Chinese Academy of Medical Science, for her generous gift of the heterozygous *Alkbh5* mice. We would like to thank Home for Researchers (www.home-forresearchers.com) for paper writing. We would also like to thank Editage (www.editage.cn) for English language editing.”

In the published article, we neglected to add an accession number and repository in the **Data Availability Statement**. The missing repository is “NCBI GEO” and the accession ID is “GSE186217.” The updated **Data Availability Statement** appears below.

“The datasets presented in this study can be found in online repositories. The names of the repository/repositories and accession number(s) can be found below: BioProject: PRJNA761579, SRA accession: SRP336482, GEO: GSE186217.”

The authors apologize for these errors and state that this does not change the scientific conclusions of the article in any way. The original article has been updated.

