# Supplementary material for: Comprehensive Analysis of the Transcriptome-Wide m6A Methylation in Mouse Pachytene Spermatocytes and Round Spermatids
Source: Front Genet. 2022 Mar 17;13:832677. doi: 10.3389/fgene.2022.832677 (PMC8968445; doi:10.3389/fgene.2022.832677)
Supplement: Supplementary file 3 [file Table1.DOCX]

**Supplementary Table 1** Sequences of primers used for qRT-PCR analysis.

| Name | Sequences |  | Product size(bp) |
| --- | --- | --- | --- |
| Mroh4 | F :5' TCTATGGCATGATCCTGCGTG3' | R :5' CAGAAACTTGGTACGGCCCAG3' | 191 |
| Prm1 | F :5' CCGTCGCAGACGAAGATGTC 3' | R :5' CACCTTATGGTGTATGAGCGG 3' | 96 |
| Lrrc71 | F: 5' CAAGTGAGCCAAGCACTACAG 3' | R: 5'TGTCTTATCTTTAGCAGCCCGAT3' | 110 |
| Dbil5 | F: 5' CCCAGGGCGACTGTAACATC3' | R: 5' GCAATGTAGATCCTCATGGCAT3' | 121 |
| H1fnt | F: 5' GGCGCAGAACTTACGATCCA 3' | R:5' GACTTCCCCTCGTGGTGAG 3' | 215 |
| Catsperd | F: 5' ACACACACAAGCATCTACTTTGA 3' | R:5' AGAGGACTGCACATTCACTGT 3' | 117 |
| Odf4 | F:5'ATGGAACCTGACTTGAATGAGGA3' | R: 5'CATTGAAAGGGCAATAGGGAGTT 3' | 101 |
| β-Actin | F: 5' GGCTGTATTCCCCTCCATCG 3' | R:5' CCAGTTGGTAACAATGCCATGT3' | 154 |
