# Supplementary material for: Comprehensive Analysis of the Transcriptome-Wide m6A Methylation in Mouse Pachytene Spermatocytes and Round Spermatids
Source: Front Genet. 2022 Mar 17;13:832677. doi: 10.3389/fgene.2022.832677 (PMC8968445; doi:10.3389/fgene.2022.832677)
Supplement: Supplementary file 4 [file DataSheet1.DOCX]

**Supplementary Figure 1**


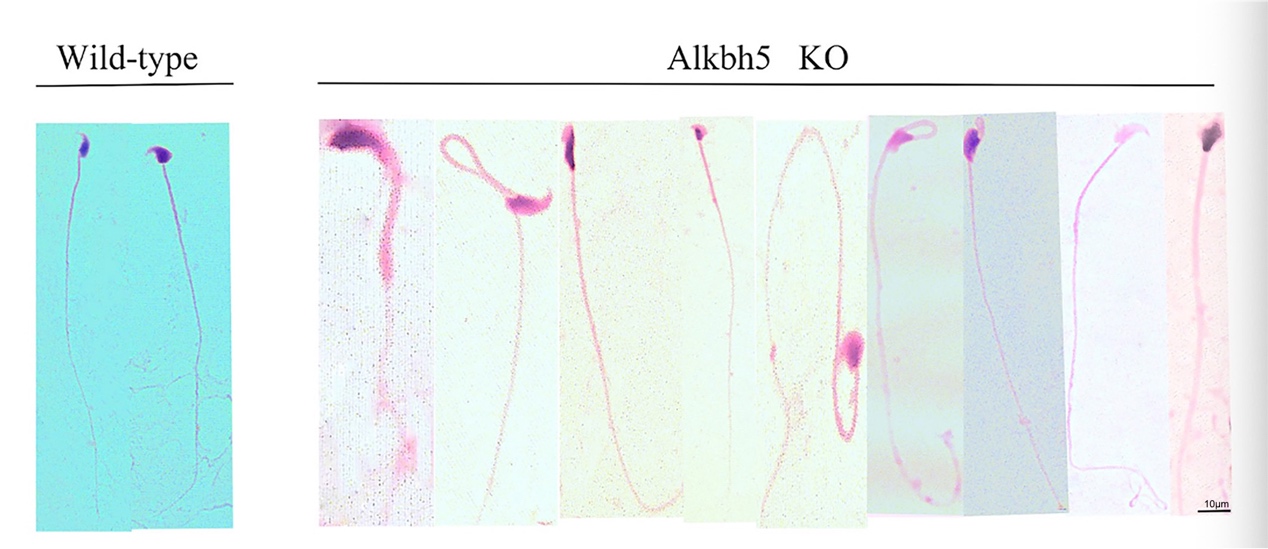


**Supplementary Figure 2**


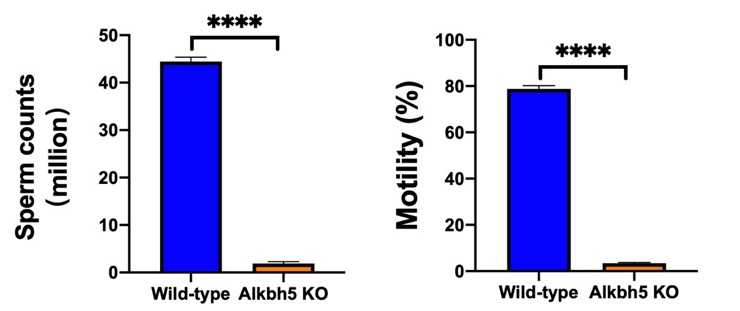


**Supplementary Figure 3**

**
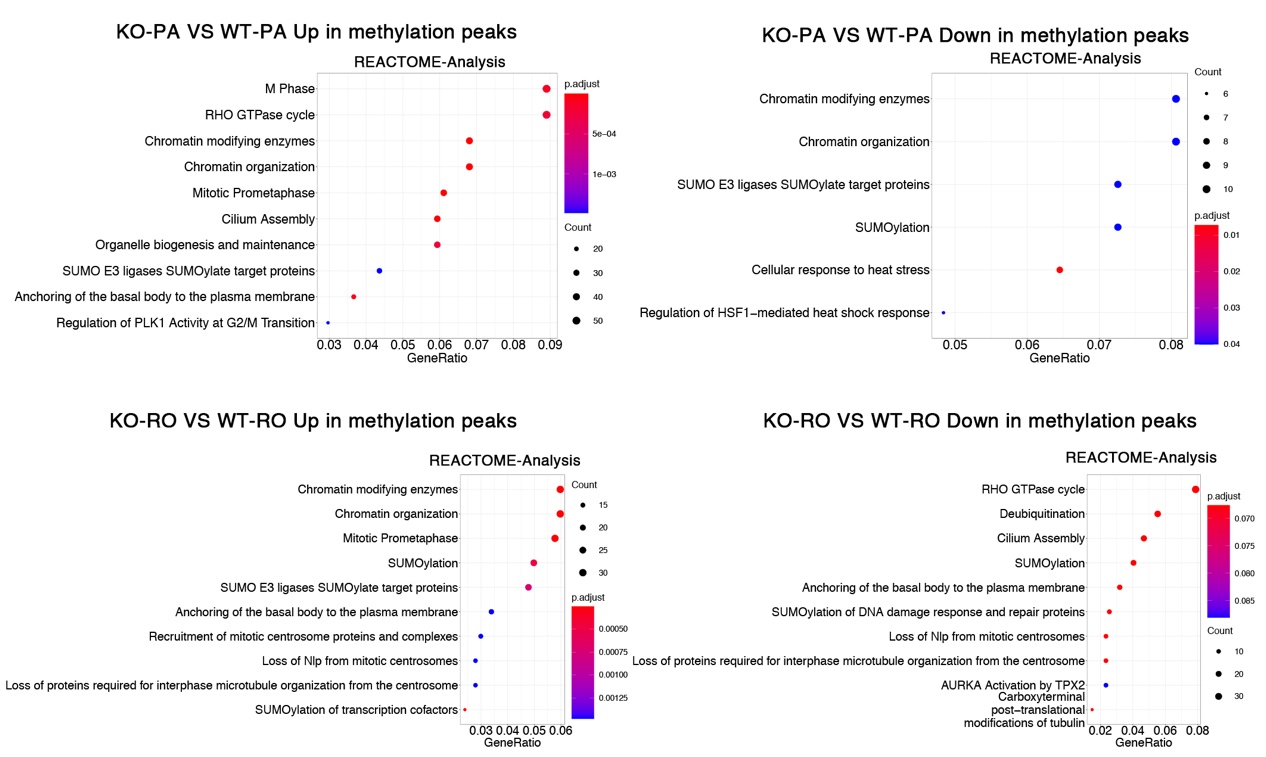
**

**Supplementary Figure 4**

**
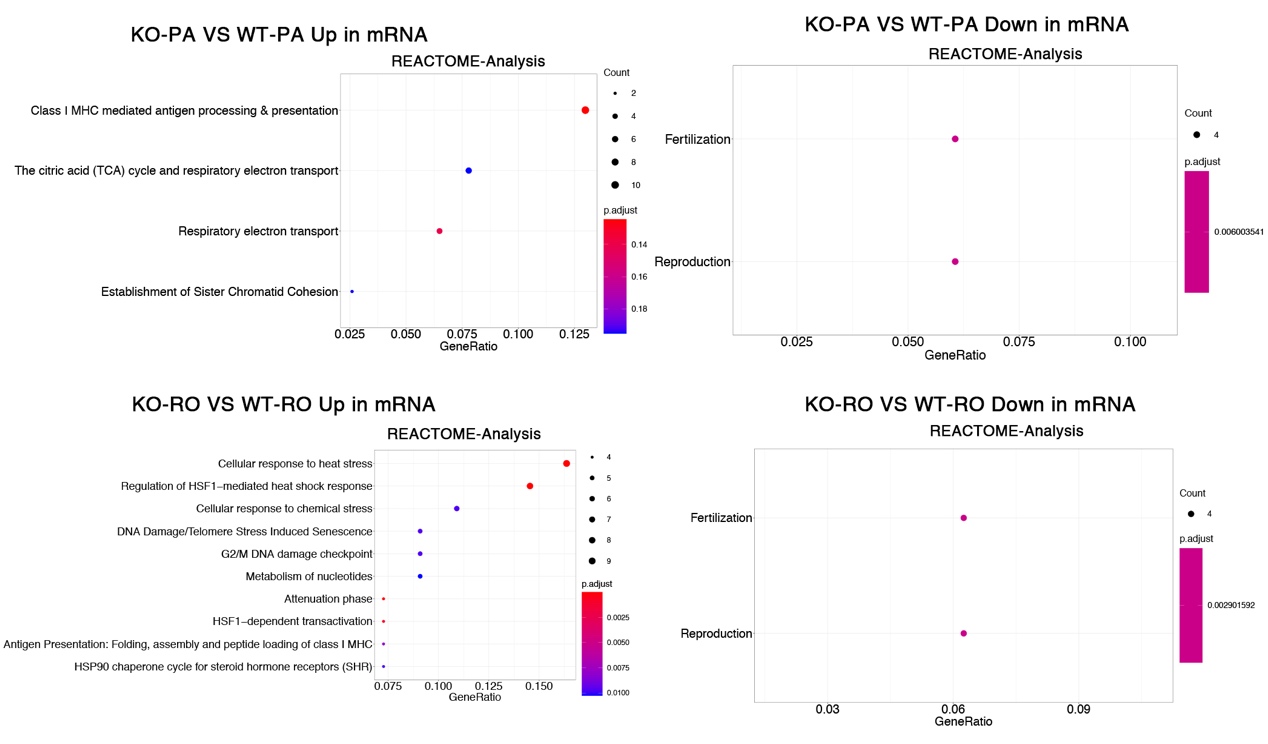
**

**Supplementary Figure 1.**the spermatozoa collected from WT and Alkbh5 KO epididymides stained with H&E. All images were captured at the same magnification (scale bars, 10 μm).

**Supplementary Figure 2.** Significant differences in sperm counts and motility between WT and Alkbh5 KO mice. Bars indicate means ± SEM (n = 5, ****p < 0.0001).

**Supplementary Figure 3.** REACTOME enrichment analysis of upregulated and downregulated methylation peaks in PA and RO between WT and Alkbh5 KO mice.

**Supplementary Figure 4.** REACTOME enrichment analysis of upregulated and downregulated mRNAs in PA and RO between WT and Alkbh5 KO mice.
